# Transient Bilateral Sixth Nerve Palsy: A Rare Sequela of Head Trauma

**DOI:** 10.7759/cureus.12805

**Published:** 2021-01-20

**Authors:** Sukainah Alalwi, Amal Alabbadi, Abdullatif M Alomair, Dunya Alfaraj

**Affiliations:** 1 Medicine, Imam Abdulrahman Bin Faisal University, Dammam, SAU; 2 Emergency, King Fahd Hospital of the University, Imam Abdulrahman Bin Faisal University, Dammam, SAU

**Keywords:** head trauma, abducens nerve, transient, palsy

## Abstract

Sixth cranial nerve palsy is uncommon but a well-recognized consequence of head trauma. Although few cases documented traumatic persistent bilateral sixth nerve palsy, a transient episode is extremely rare and not reported before. We report an unusual case of transient bilateral abducens nerve palsy after minor head trauma, which was completely resolved without any intervention after 20 min. Diplopia may relate to anatomical lesion, and even transient diplopia can guide us to a serious anatomical lesion that may occur. CT scan of the head revealed a left nondisplaced occipital fracture, which extends to the left petrous bone. The patient was admitted for 24-h observation and discharged home on paracetamol. This case emphasizes the need to recognize the rare sequences of minor head trauma and manage them appropriately.

## Introduction

The abducens nerve is the sixth cranial nerve that is responsible for ipsilateral eye abduction [1]. Post-traumatic sixth nerve palsy is usually a result of severe head or facial trauma and is associated with loss of consciousness. This may occur in the presence or absence of a skull base or cervical fracture. Unilateral abducens nerve palsy occurs in 1%-2.7% of all head traumas. However, bilateral palsy is extremely rare and is mostly associated with additional intracranial or spinal injury [2-3]. If the damage involved the nerve bilaterally, this usually would present with persistent diplopia, large-angle esotropia, and severe restrictions of abduction [4]. We report an extremely rare case of transient bilateral sixth nerve palsy following head trauma that resolved immediately after 20 min since the symptoms onset. 

## Case presentation

A 21-year-old female presented to the ED with a history of headache and diplopia after she hit her head on the edge of the table. Immediately following the accident, the patient had a transient loss of consciousness. After about two minutes, she regained consciousness but was confused and complained of headache, blurred and double vision. In addition, the family noticed abnormality in the patient’s eye and they described it as squint. 

The headache started suddenly and it was continuous. It was in the back of the head and neck radiating to the front of the head around the eyes. The pain was dull aching in nature with a severity of seven out of ten. It was aggravated by light, but there were no relieving factors. This was associated with diplopia, which lasted for about 10 min. The patient denied any history of change in balance, difficulty in speech, weakness, numbness, deficit of memory, taste changes, seizures, irritation, vomiting, and nausea. Past medical, surgical, social, medication, and allergy history were unremarkable. Systemic review was unremarkable.

**Figure 1:**
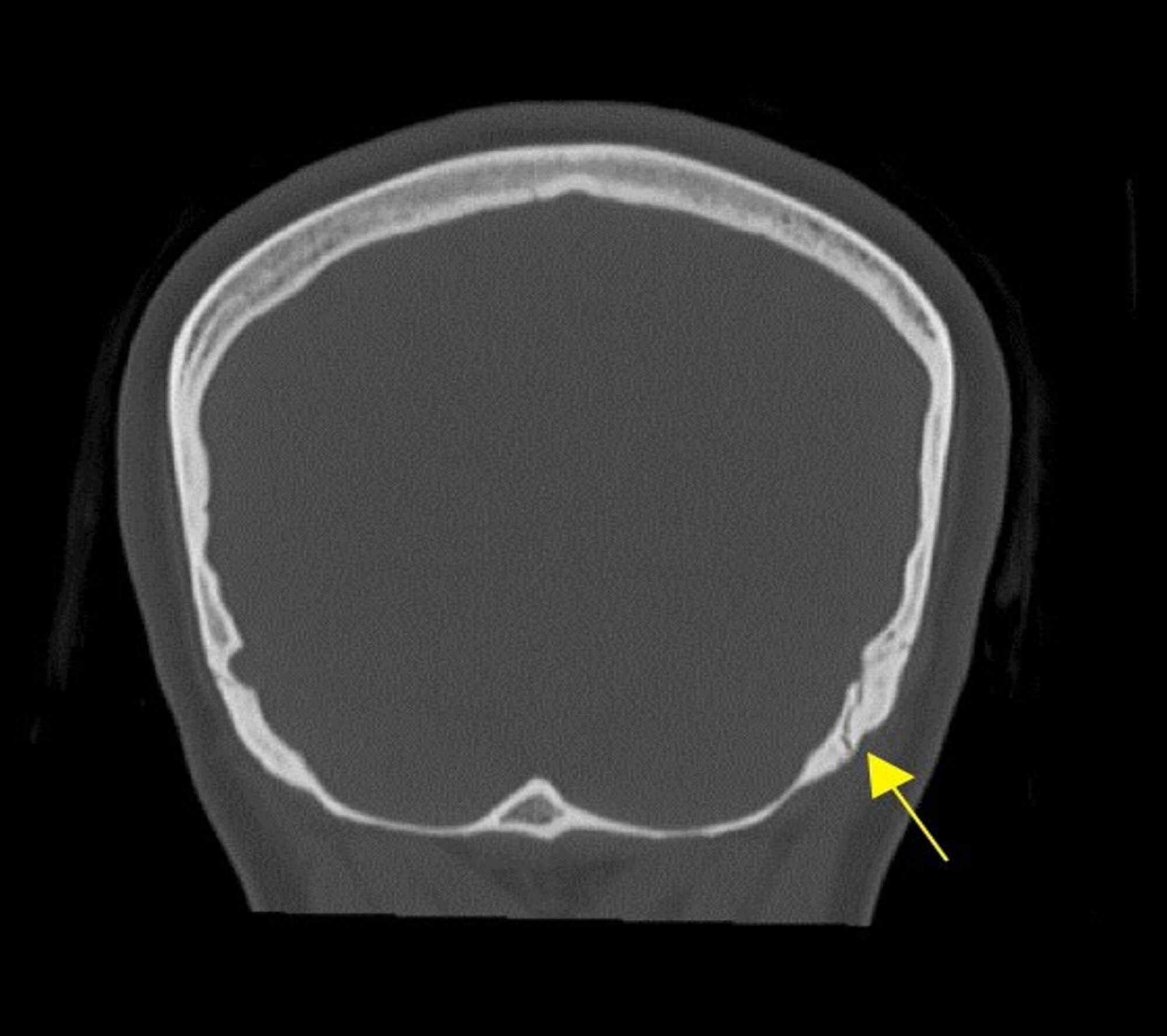
Coronal view of nonenhanced head CT showing the left occipital bone fracture.

**Figure 2:**
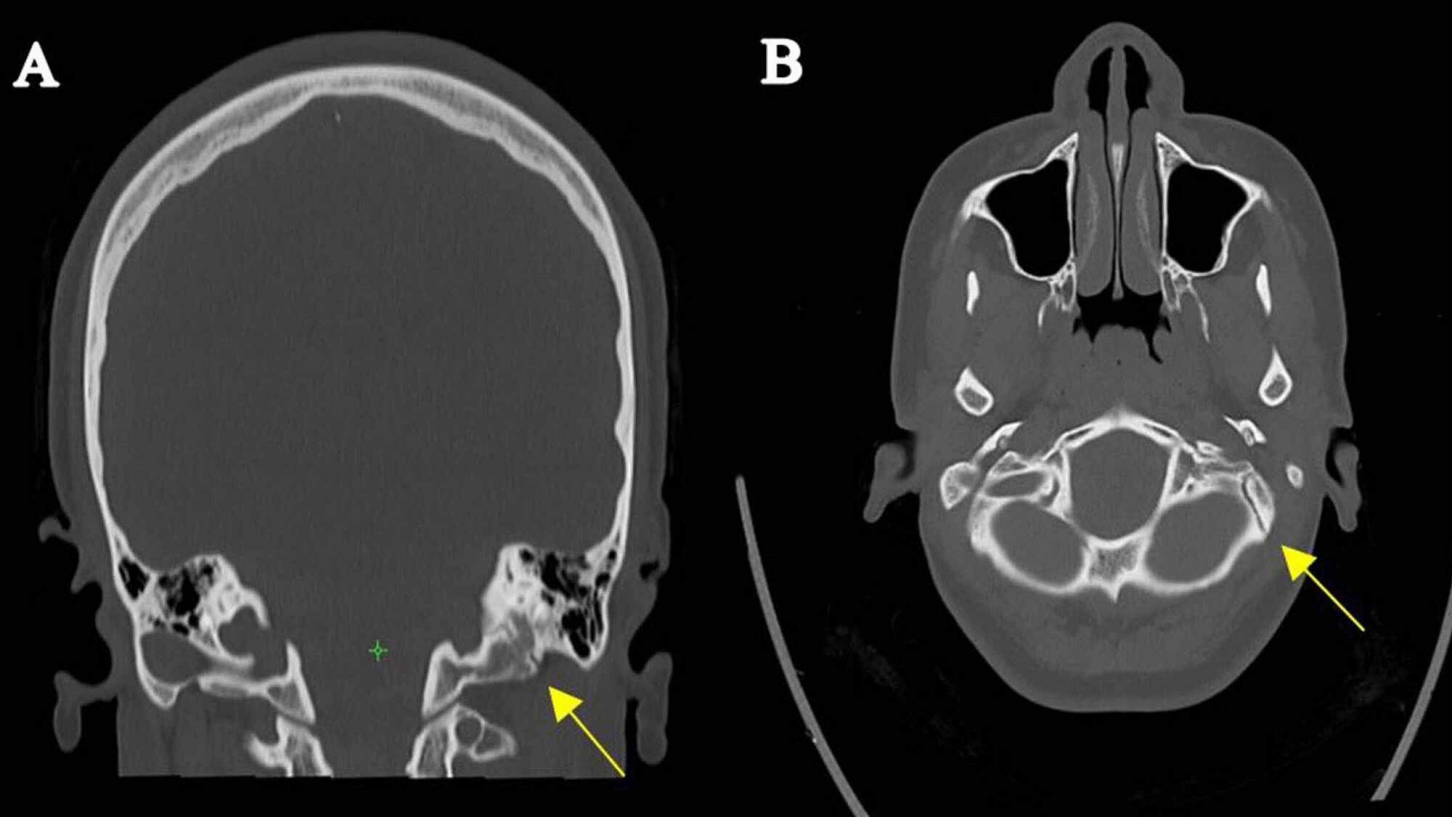
Nonenhanced head CT showing the left petrous bone fracture. (A, coronal view; B, axial view).

Upon hospital arrival, her Glasgow Coma Scale (GCS) was 15/15. Neurological examination was conducted, and it showed bilateral lateral gaze palsy which was completely resolved without any intervention after 20 min since the symptoms started. The patient spent 10 min to arrive to the hospital, and 10 min in the ED before the resolution of the symptoms. No other significant findings were recorded in general physical and neurological examination. Nonenhanced CT scan of the head was done, and it showed a nondisplaced fracture over the left occipital bone (as shown in Figure 1), which extends to the left petrous bone as a transverse fracture (as shown in Figure 2). 

The patient was admitted for 24-h observation unit and given 1 g IV paracetamol infusion for her headache. She was discharged home from the observation unit on oral paracetamol 500 mg every 6 h per needed, and with a follow-up appointment at the neurosurgery clinic after two weeks. After that, the patient lost to follow-up.

## Discussion

Anatomically, the abducens nerve has a long and delicate route passing through five regions: brainstem, subarachnoid space, petrous apex, cavernous sinus, and orbit. As the nerve exits the pons, where its nucleus located, it ascends vertically through the subarachnoid space. After 15 mm, it ascends over the ridge of the petrous bone and passes under the petroclinoidal (Gruber’s) ligament. This space is called Dorello’s canal, which is defined by the petrous bone apex, the posterior clinoidal process, and Gruber’s ligament. After that, the nerve passes through the cavernous sinus and the superior orbital fissure to innervate the lateral rectus muscle [3]. 

The frequently affected locations for damage are the points of the abducens nerve entry into the extradural space and the petrous ridge. The vulnerability of this nerve to stretching and downward displacement by trauma is related to its tortuous long intracranial route, as well as its strong dural attachments in the cavernous sinus. This displacement against the petrous bone causes contusion and edema [5-6]. Anatomically, it has a close relation to several brain structures, which makes its injury associated with cranial nerves III, IV, V, and VII palsies [7]. 

As mentioned earlier, bilateral sixth nerve palsy after mild head trauma rarely occurs. Most commonly, it results from a vascular origin, inflammatory diseases, and tumors. Thus, few cases of post-traumatic bilateral sixth nerve palsy have been reported in the literature [8]. 

Patients with abducens nerve palsy commonly present with: binocular horizontal diplopia, esotropia in primary gaze position which become worse in lateral gaze position toward the palsied eye, and abnormal posture of the head with the face turned toward the direction of the paretic muscle. In mild cases, patients may complain of dizziness, difficulty focusing, and blurred vision [1, 9]. 

The evaluation of sixth nerve palsy clinically and radiologically depends on patient’s age and whether the palsy is isolated or associated with other neurological findings. Patients younger than 45 years with sixth nerve palsy will require a neurological workup even in isolated palsy cases [9]. Neuroimaging evaluation is indicated in most patients. A central lesion might be detected on MRI or CT scan. However, MRI is superior to CT in imaging the posterior fossa and detecting subacute minimal contusion hemorrhages [10]. 

The Canadian CT Head Rule was developed for patients with minor head injuries, based on strong evidence. Minor head injury can be defined as: definite amnesia, witnessed loss of consciousness, or disorientation in a patient with a GCS score of 13-15. This rule divides the patients into high and medium risk groups based on seven clinical variables [11]. When applying this rule to our case, there was no indication for head CT scan. However, our patient presented with unusual and rare manifestation, and one of the most frequently affected locations in case of sixth nerve palsy is the entry point of the petrous ridge, which is a part of the skull base. Therefore, head CT was done to look for a possible basal skull fracture. Although no signs of basal skull fracture were detected in this case, head CT showed left nondisplaced occipital fracture that extends to the left petrous bone. This confirms our suspicion of basal skull fracture and explains the reason for abducens nerve palsy. Here, the question that could be raised in this context: is transient sixth nerve palsy considered a sign of basilar skull fracture and should be indicated for head CT even if there were no other indications?

Table 1 contains a sample of selected studies discussing head CT findings of five reported cases with post-traumatic isolated bilateral abducens nerve palsy. In contrast to our case, all the patients presented with persistent abducens nerve palsy in which they required treatment with follow-up appointments. The recovery period in these cases ranges from a few months up to one year, and three of them required corrective surgery. 

**Table 1:** A summary of five reported cases on post-traumatic isolated bilateral abducens nerve palsy describing the head CT findings.

Author	No. of cases	Head CT findings
Advani et al. (2003) [3]	1	Normal
Czyz et al. (2011) [12]	1	Normal
Jaiswal et al. (2014) [8]	1	Normal
Fam et al. (2015) [13]	1	Posterior wall fracture of the left maxillary sinus Multiple hyperdensity foci in left frontal and right corona radiata Left frontal subdural hygroma
Serio et al. (2019) [14]	1	Normal

A dilated fundus examination is required for all patients to rule out papilledema which results from increased intracranial pressure, and to look for retinal vascular change associated with diabetes and hypertension if indicated [9]. 

Spontaneous recovery is common in patients with isolated nontraumatic and traumatic sixth nerve palsy [1]. However, the deficit is usually not completely resolved in patients with a skull base fracture [5]. The spontaneous recovery rate of traumatic unilateral and bilateral sixth nerve palsy is 84% and 38%, respectively [6]. Initial conservative treatment includes occlusion patch and Fresnel prisms. If no significant improvement is seen, Botulinum toxin injection is used to cause a temporary muscle paralysis allowing for single vision [7]. This case is particularly interesting because there was spontaneous recovery after a brief period without any intervention. 

## Conclusions

Isolated transient bilateral abducens nerve palsy is extremely rare after a minor head injury. Neurological examination should be given special consideration to rule out other cranial nerve involvement. The presence of bilateral palsy is usually associated with severe trauma and should raise a high index of suspicion for associated injuries; thus, head CT must be obtained.
